# Worldwide continuous gap-filled MODIS land surface temperature dataset

**DOI:** 10.1038/s41597-021-00861-7

**Published:** 2021-03-04

**Authors:** Shilo Shiff, David Helman, Itamar M. Lensky

**Affiliations:** 1 Department of Geography and Environment, Bar-Ilan University, Ramat Gan, Israel; 2 Institute of Environmental Sciences, Soil and Water Sciences Unit, The Robert H. Smith Faculty of Agriculture, Food and Environment, The Hebrew University of Jerusalem, Rehovot, Israel; 3 Advanced School for Environmental Sciences, The Hebrew University of Jerusalem, Jerusalem, Israel

**Keywords:** Atmospheric science, Environmental sciences

## Abstract

Satellite land surface temperature (LST) is vital for climatological and environmental studies. However, LST datasets are not continuous in time and space mainly due to cloud cover. Here we combine LST with Climate Forecast System Version 2 (CFSv2) modeled temperatures to derive a continuous gap filled global LST dataset at a spatial resolution of 1 km. Temporal Fourier analysis is used to derive the seasonality (climatology) on a pixel-by-pixel basis, for LST and CFSv2 temperatures. Gaps are filled by adding the CFSv2 temperature anomaly to climatological LST. The accuracy is evaluated in nine regions across the globe using cloud-free LST (mean values: R^2^ = 0.93, Root Mean Square Error (RMSE) = 2.7 °C, Mean Absolute Error (MAE) = 2.1 °C). The provided dataset contains day, night, and daily mean LST for the Eastern Mediterranean. We provide a Google Earth Engine code and a web app that generates gap filled LST in any part of the world, alongside a pixel-based evaluation of the data in terms of MAE, RMSE and Pearson’s *r*.

## Abstract (summary)

**Table:** 

Measurement(s)	temperature of air • land surface temperature
Technology Type(s)	satellite imaging of a planet • computational modeling technique
Factor Type(s)	year of data collection • daytime and nighttime surface temperature bands
Sample Characteristic - Environment	climate system
Sample Characteristic - Location	Eastern Mediterranean

Machine-accessible metadata file describing the reported data: 10.6084/m9.figshare.14035028

## Background & Summary

Land Surface Temperature (LST) is a key variable in surface energy and water balances, as well as in climatological and environmental studies such as agriculture^1–3^, epidemiology^4–6^, and ecology^7–9^.

Land and air surface temperatures can be derived from *in-situ* measurements, satellite observations (LST) and numerical weather prediction (NWP) models. Meteorological stations provide continuous air temperature data (usually at 2 m above the ground), but the usefulness of such data depends on the geographical distribution in terms of location and density of the stations. NWP models are good at depicting weather conditions, defined as the temperature deviation (anomaly) from the seasonal mean temperature (climatology). NWP models are not limited by cloud interference (as opposed to satellite observations), but the surface properties in NWP are roughly considered. Sensors onboard polar-orbiting satellites, such as the Moderate Resolution Imaging Spectroradiometer (MODIS), produce daily, almost global coverage of LST observations at a spatial resolution of 1 km. However, these observations are limited by cloudy conditions. Gap filled LST products (Level 4 analyses) are traditionally generated using satellite data from several sensors at a spatial resolution of 0.05° ^10^. For many applications (such as agricultural applications^1,9^) a finer resolution of 1 km is required. In many cases, 1-km is not enough and sharpening/fusion methods^11–13^ are used to produce LST data at a much finer spatial resolution (~30 meters).

Time series analysis of LST can provide the climatological seasonal behavior of LST at the topoclimate scale^14,15^, as well as the seasonal effect of vegetation and soil properties^16^. The atmospheric circulation at the meso and synoptic scales has a significant impact on LST^15,17^. The atmospheric circulation at the synoptic-scale has also a significant effect on the temperature difference between LST and the 2 m air temperature^18^.

Global daily datasets of LST suffer from missing data due to pixels that are overcast by clouds. Several methods were suggested for estimating LST of cloudy pixels, ranging from simple to highly complex. Simple methods include the use of spatial interpolation (i.e. using data from nearby pixels to retrieve LST in pixels with missing data) and time interpolation techniques (using available data from earlier observations), as well as air temperature to surface temperature relationships^19,20^. Methods often include cubic spline interpolation^16^ and surface energy balance^21^ to reconstruct poor-quality and invalid LST in MODIS pixels. Most of these methods, however, do not consider the influences of surface vegetation/soil properties on the surface temperature, which makes their transferability to other areas less reliable.

More complex methods (those including various datasets and advanced statistical methods) include the use of data from meteorological stations and a “multiplier function” that depends on satellite-based normalized difference vegetation index (NDVI)^22^, singular spectrum analysis^23^, or a combined polar-orbiting thermal infrared and passive microwave (PMW) data^24^. While these methods are more promising in terms of spatial transferability, their complexity limits their use mostly to the remote sensing research community. A simple yet reliable gap-filling method that uses freely available global datasets on an easily accessed platform could benefit users relying on spatially and temporally continuous temperature data.

Here we use a simple method that combines the 1-km LST product of MODIS^25^ with the 0.2 arc degrees^26^ modeled surface temperature from the National Centers for Environmental Prediction (NCEP) Climate Forecast System Version 2 (CFSv2) to provide a spatiotemporally continuous gap filled LST at the original 1-km resolution of MODIS. The dataset is offered globally and can be simply derived through the Google Earth Engine (GEE) platform without needing to download many datasets to the user’s own computer. The JavaScrip code for GEE is provided to generate the data everywhere around the globe, test the data, and validate it against observed LST. In addition, a full dataset is provided for the Eastern Mediterranean that include day, night, and daily mean gapfilled LST for 2002–2020.

## Methods

### Google earth engine platform

GEE is a parallel computation service platform for advanced image analysis that hosts a variety of remote sensing and geospatial datasets^27^. GEE leverages Google’s cloud computing services with analytical capabilities that are otherwise heavy consumers of time and computation resources. Since our research uses a time series of more than 18 years of daily datasets, we chose GEE as our research platform. Furthermore, GEE helps researchers to easily disseminate their products, enabling the provision of codes and web apps alongside ready-to-use datasets for the benefit of the scientific community.

### Study area for validation

**Fig. 1:**
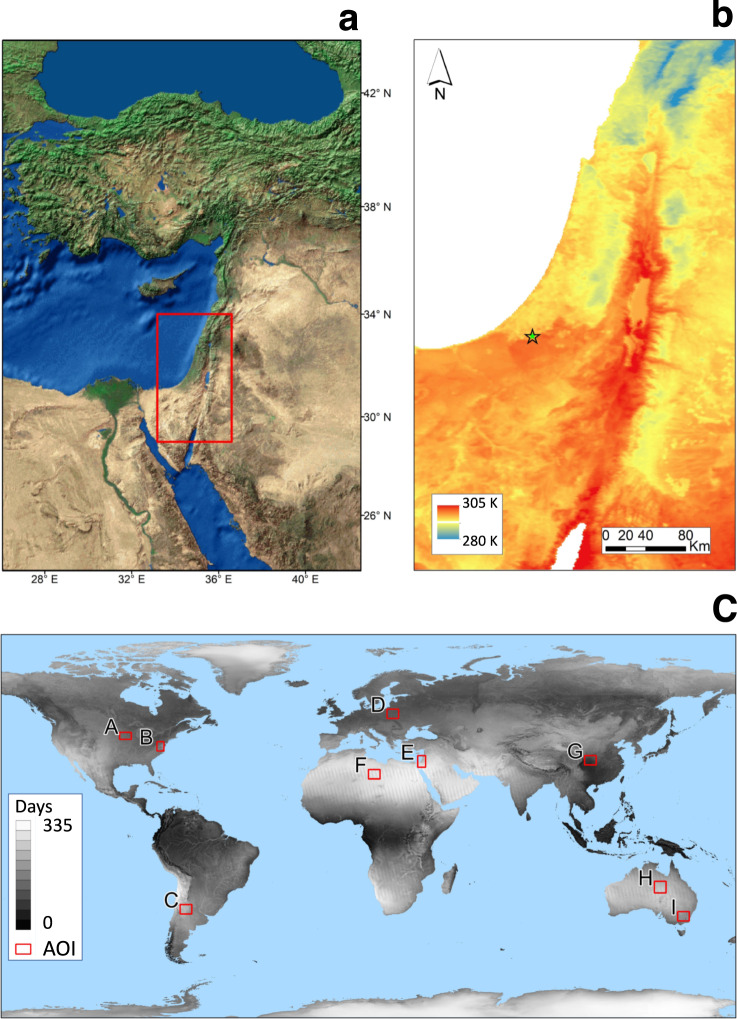
Study area and validation sites. (**a**) The study area in the Eastern Mediterranean (covering an area of 175,000 km^2^) and (**b**) the mean daily LST (in kelvin degrees) for this area (2002–2019, 6329 days). The green star in b indicates the location of the pixel from which the time series for Fig. 2 were extracted. (**c**) The average number of clear sky days per year for 2002–2019 (1–365) is displayed in grey scale, the red rectangles represent the location of the nine global validation sites (Table 2), including the study area (E).

The study area, located in the Eastern Mediterranean (Fig. 1a,b), was selected because of the high spatial variability of climatic conditions^14^. This variability is due to the region’s complex orography (with elevations from −430 up to 2814 m) and the spatial heterogeneity of land covers. Eight additional regions were selected for validation, covering six continents (Fig. 1c).

### Satellite and numerical weather prediction model data

We used the level 3 MODIS LST^28^ product (MYD11A1 Version 6) from the Aqua polar-orbiting NASA sun-synchronous satellite (1:30 AM/PM local time). MYD11A1 provides daily LST and Emissivity at 1 km spatial resolution in a 1,200 by 1,200 km grid (fixed tiles). The pixel temperature value is derived from the MYD11_L2 swath product. Above 30 degrees latitude, some pixels may have multiple observations where the criteria for clear sky are met. When this occurs, the pixel value is a result of the average of all qualifying observations. Provided along with the daytime and nighttime surface temperature bands are associated quality control assessments, observation times, view zenith angles, and clear-sky coverages. We used 2002–2019 LST data to retrieve the seasonal behavior of LST at cloud-free conditions. The yearly average of the number of days with cloud-free MODIS LST data at any given location is shown in Fig. 1c.

To complement MODIS LST, we used the surface air temperature derived from the NCEP CFSv2 model^26^. CFSv2 surface air temperature is calculated at 2 m above the ground at a spatial resolution of 0.2°. We chose CFSv2 because of its relatively high spatial resolution (compared to, for e.g., ERA-40 and NCEP/NCAR reanalysis products of 125 km and 2.5°, respectively), its temporal coverage (6 hourly product), and because historic CFSv2 data is freely availabile on the GEE platform, which can be easily accessed and used even by non-climate researchers. One significant drawback, however, is the distinct physical meaning of the two temperature products (i.e., LST from MODIS and 2-m temperature from CFSv2). However, while CFSv2 does provide a skin temperature product, it is less reliable compared to the 2-m temperature product because skin temperature usually varies with surface characteristics (e.g., land cover), which are not well captured by numerical models.

The NCEP CFSv2 is based on a fully coupled global NCEP Reanalysis model representing the interaction between the Earth’s atmosphere, oceans, land, and sea ice for the period 1979–2011^26,29^. CFSv2 provides reforecasts that are initialized four times per day (0000, 0600, 1200, and 1800 UTC). These are effectively the first guess fields that are the basis of an operational analysis or a reanalysis. Therefore, it is strictly a model product which is not informed by the most recent observations (ruling out circularity), but since it is run within the context of a data assimilation system it does carry the memory of previous observations. The use of a short range (6 hour) forecast is based on the assumption that the model error or drift is minimal over this period. The data is available from 1979 until present. Both products (MYD11A1 and CFSv2) are available on the GEE platform.

### Calculating the climatology and anomaly of satellite and model data

We used Temporal Fourier Analysis (TFA) to derive the climatological temperatures of the cloud-free satellite (MODIS LST) and CSFv2 temperatures. The TFA describes the seasonal cycles of temperature in terms of annual, bi-annual and tri-annual components (or ‘harmonics’), each described by its phase and amplitude. These Fourier harmonics may be recombined, providing a smoothed signal, which is regarded here as the climatological expected temperatures:

1 $$LS{T}_{clim}\left(t\right)=\bar{LST}+{\sum }_{i=1}^{n}\,{A}_{i}cos({\omega }_{i}t-{\varphi }_{i}),$$

*LST*_*clim*_(*t*) is the climatological MODIS LST at Julian day *t*; $\bar{LST}$ is the mean annual LST, *A*_*i*_ is the amplitude of the *i*^th^ harmonic component, while *n* is the number of harmonic components. We used here the first three harmonics (*n*=3), following Scharlemann *et al*.^30^ and Lensky and Dayan^14^. *φ*_*i*_ is the phase and *ω*_*i*_ is the frequency (*ω*_*i*_ = 2*πi*/365) of the *i*^th^ harmonic component. TFA was applied on MODIS LST to derive the climatological LST and on CSFv2 to derive the climatological temperature from which the anomaly was calculated (*T*_*anom*_, the deviation of the actual temperature from the climatological temperature). To calculate *A*_*i*_ and *φ*_*i*_ we used time series of one year (365 Julian days) with the 2002–2019 mean clear sky LST data for each Julian day. This enabled estimating mean LST for pixels having a few clear sky days in the 2002–2019 time series as well (such as at equatorial regions).

LST under cloudy conditions may be higher or lower than under clear sky conditions^31^. This may be attributed to atmospheric circulation through positive or negative temperature advection, or to change in the radiative balance, e.g. by blocking shortwave radiation at daytime or by blocking the emitted longwave radiation at nighttime. These effects are taken into account in NWP models such as CSFv2. The location of the clouds in NWP models at resolution of 0.2° is not comparable to satellite observations, nevertheless, this LST product aims to provide min/max daily LST, which depends on the amount and duration of cloud cover, that is taken into account in NWP. Therefore, CFSv2 is a good data source for filling LST gaps. Moreover, while gap-filling algorithms that use spatial interpolation emulates clear-sky LST, *LST*_*cont*_ represents the actual LST under the cloud (as in PMW retrievals).

### Combining the satellite and model temperatures

Surface temperature at a specific time and date can be regarded as composed of two components: (a) the long term mean of the temperature at that specific time and date (climatology), and (b) the deviation from that mean due to the weather (anomaly). The climatological temperature is determined mainly by the Earth’s changing position with regard to the Sun, having a seasonal pattern that can be inferred using harmonic analysis (e.g. TFA). The anomaly is determined mostly by the synoptic-scale circulation and can be inferred from circulation models at coarse spatial resolution.

To estimate the actual LST at time *t* (*LST*_*cont*_(*t*)) we add the CSFv2 temperature anomaly (*T*_*anom*_(*t*)) to the fine-scale (1 km) observed (MODIS) climatological LST (*LST*_*clim*_(*t*)):

2 $$LS{T}_{cont}\left(t\right)=LS{T}_{clim}\left(t\right)+{T}_{anom}\left(t\right)$$

The actual clear sky satellite observations (MODIS LST) are used in the dataset whenever they are available. We use *LST*_*cont*_ only to estimate the missing LST data (cloudy pixels). The relationship between LST and 2 m air temperature is not globally consistent, nevertheless we use CFSv2 data only for cloudy conditions in which LST and 2 m air temperature are often close (e.g., within 2 °C)^32^.

*T*_*anom*_ was calculated as the daily average of the four outputs: 0000, 0600, 1200, and 1800 UTC, which allows to match between the sun synchronous (local time) MODIS observations and the model outputs at coordinated universal time (UTC). The MODIS LST product at 1.30 am/pm is close to the minimum/maximum diurnal LST. Accordingly, in the study area we used CFSv2 at 00/12UTC, which is close to the minimum/maximum diurnal temperature (02:00/14:00 local time in the Eastern Mediterranean), to produce our nighttime and daytime *LST*_*cont*_ products. We used CFSv2 over the 24 hours (i.e. 00, 06, 12, and 18 UTC) to produce the average daily *LST*_*cont*_. The daily product as described here is used globally, while the gap-filed day and night products (provided in the GEE application and code) uses *LST*_*clim*_ (and not *T*_*anom*_).

**Fig. 2:**
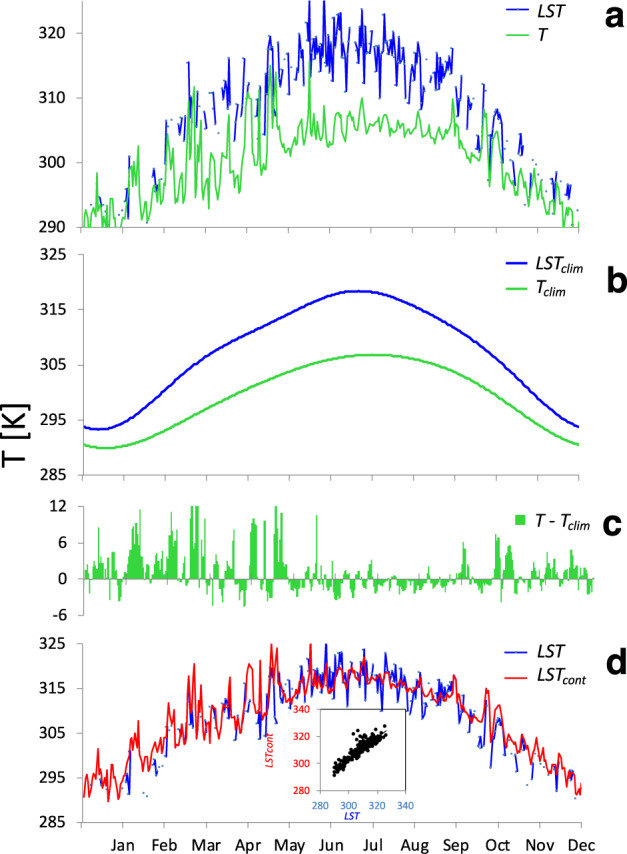
Methodology for production of continuous LST (*LST*_*cont*_). (**a**) Day (MODIS) *LST* and CFSv2 2-m air temperature (*T*) of one year (2018); (**b**) their climatological values (*LST*_*clim*_ and *T*_*clim*_), (**c**) CFSv2 temperature anomaly (*T-T*_*clim*_); and (**d**) *LST*_*cont*_ for a single pixel (green star in Fig. 1b). Insert shows the regression of *LST*_*cont*_ vs. *LST* (*r* = 0.9147, *p* < 0.0001, n = 245 days).

Figure 2a shows an example of the original time series of MODIS LST and CSFv2 surface temperature in a single pixel in the study area (green star in Fig. 1b), and their corresponding climatological temperatures (Fig. 2b). The calculated *T*_*anom*_ and final *LST*_*cont*_ product are presented in Fig. 2c,d, respectively.

## Data Records

The dataset is published in Zenodo^33^ at the resolution of the MYD11A1 product (~1 Km) and consists of two sets of files: (a) geo-located daily continuous LST (Day, Night and Daily mean) and (b) validation (MAE, RMSE and Pearson (*r*)) for the same domain, on a yearly basis. The spatial domain of the data is located on the Eastern Mediterranean as described in Fig. 1a.

(a) In the first set of files, we provide *LST*_*cont*_ - a continuous gap-filled LST dataset at 1 km spatial resolution, as described in this paper. Data are stored in GeoTIFF format as signed 16-bit integers using a scale factor of 0.02, with one file per day, each defined by 3 dimensions (day, night, and daily average *LST*_*cont*_). File names follow this naming convention: LST_ <YYYY_MM_DD> .tif, where <YYYY> represents the year, <MM> represents the month and <DD> represents the day. Files of each year (2002–2020) are compressed in a ZIP file. This dataset is also provided in NetCDF format.

(b) The second set of files contain the validation dataset (LSTcont_validation.tif) in which the MAE, RMSE, and Pearson (*r*) of the validation with observed LST are provided. Data are stored in GeoTIFF format as signed 32-bit floats, with the same spatial extent and resolution as the dataset (a). These data are stored with one file containing three bands (MAE, RMSE and Perarson_r). The same data with the same structure is also provided in NetCDF format.

After this work was accepted, during the curation process, 2020 data were uploaded and added to the Zenodo record.

## Technical Validation

The insert in Fig. 2d shows good agreement between *LST*_*cont*_ (calculated) and the MODIS *LST* (observed) in a single pixel in the Eastern Mediterranean (green star in Fig. 1b) for the year 2018 (*r* = 0.917, *p* < 0.0001, n = 245 days).

We used cloud-free pixels of MODIS day, night and daily average *LST* in the study area for 2002–2019 to validate the model. Table 1 shows the results of this validation.

**Table 1:** Model performance metrics of day, night and daily mean LST for the Eastern Mediterranean (E in Fig. 1c and Table 2).

	MAE			RMSE			Pearson (*r*)		
	*LST*_*clim*_	*LST*_*cont*_	Δ	*LST*_*clim*_	*LST*_*cont*_	Δ	*LST*_*clim*_	*LST*_*cont*_	Δ
**Daytime**	2.909	**2.672**	0.237	3.640	**3.443**	0.197	0.941	**0.951**	0.010
**Nighttime**	**1.940**	2.022	−0.083	**2.475**	2.592	−0.117	0.939	0.939	0.001
**Daily mean**	2.229	**1.844**	0.385	2.870	**2.409**	0.461	0.952	**0.971**	0.019

The improvement of *LST*_*cont*_ over *LST*_*clim*_ in daytime and daily mean is highlighted in bold. This is not the case in nighttime where *LST*_*clim*_ performs better.

**Fig. 3:**
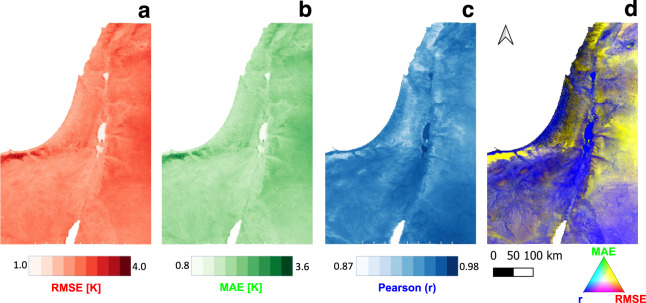
Spatial variations of the performance metrics i.e. (**a**) RMSE, (**b**) MAE, (**c**) Pearson (r) and (**d**) RGB image of all above three measures (Red – RMSE, Green – MAE, and Blue – Pearson’s-r). Blue colors represent high Pearson’s-r and low RMSE and MAE values (mostly in lower elevations), while yellow colors represent lower Pearson’s-r and higher RMSE and MAE.

*LST*_*cont*_ was validated against cloud free pixels for the entire time series (2002–2019). The spatial variations of the mean values of Root Mean Square Error (RMSE), Mean Absolute Error (MAE) and Pearson-r, of the Daily *LST*_*cont*_ in the study area are provided in Fig. 3.

At daytime, a good agreement between *LST*_*cont*_ and the observed *LST* was found (*r* = 0.951; *p* < 0.001), showing an improvement compared to *T*_*clim*_ (*r* = 0.941). At nighttime the variability of the temperatures (and anomalies) is smaller than that of daytime^32,34^. The MAE and RMSE are therefor also lower, resulting in a high correlation between *LST*_*cont*_ and MODIS *LST* (*r* = 0.939), but with no improvement over *T*_*clim*_, i.e., the contribution of the anomaly to *LST*_*cont*_ is significant at daytime, but not at nighttime. Therefore, we used daily CSFv2 anomalies but multiplied it by a factor of 1/2, which resulted in a slightly better performance of *LST*_*cont*_ compared to *T*_*clim*_ (with respect to the clear sky MODIS *LST* observations): Pearson-r of 0.971 vs. 0.952; RMSE of 2.41 °C vs. 2.87 °C; and MAE of 1.84 °C vs. 2.23 °C respectively.

Table 2 shows the statistics of the validation (performance metrics) of daily mean *LST*_*cont*_ in the nine regions in Fig. 1c. The average R^2^ was 0.93 (Each area with *p* < 0.001), with RMSE that ranges from 2.41 °C to 3.26 °C, and MAE in the range of 1.84 °C to 2.56 °C. These values are comparable to other reported gap filling methods^23,35–40^. We further conducted an additional linear regression to each of the four main seasons separately (June-Aug, Sep-Nov, Dec-Feb, Mar-May). By doing so, we were able to “clean” the seasonal, autocorrelated signal from the time series. The results of these correlations were also significant, with an average Pearson’s-r of 0.81 and RMSE of 2.31 °C (*p* = 0.01; MAE = 1.82 °C).

**Table 2:** Daily mean *LST*_*cont*_ for the areas in Fig. 1c.

	A	B	C	D	E	F	G	H	I
MAE	2.14	1.91	2.21	2.28	1.84	1.93	2.56	1.95	2.17
Pearson (r)	0.97	0.97	0.95	0.96	0.97	0.96	0.96	0.96	0.96
RMSE	2.76	2.45	2.8	2.86	2.41	2.59	3.26	2.5	2.77

**Fig. 4:**
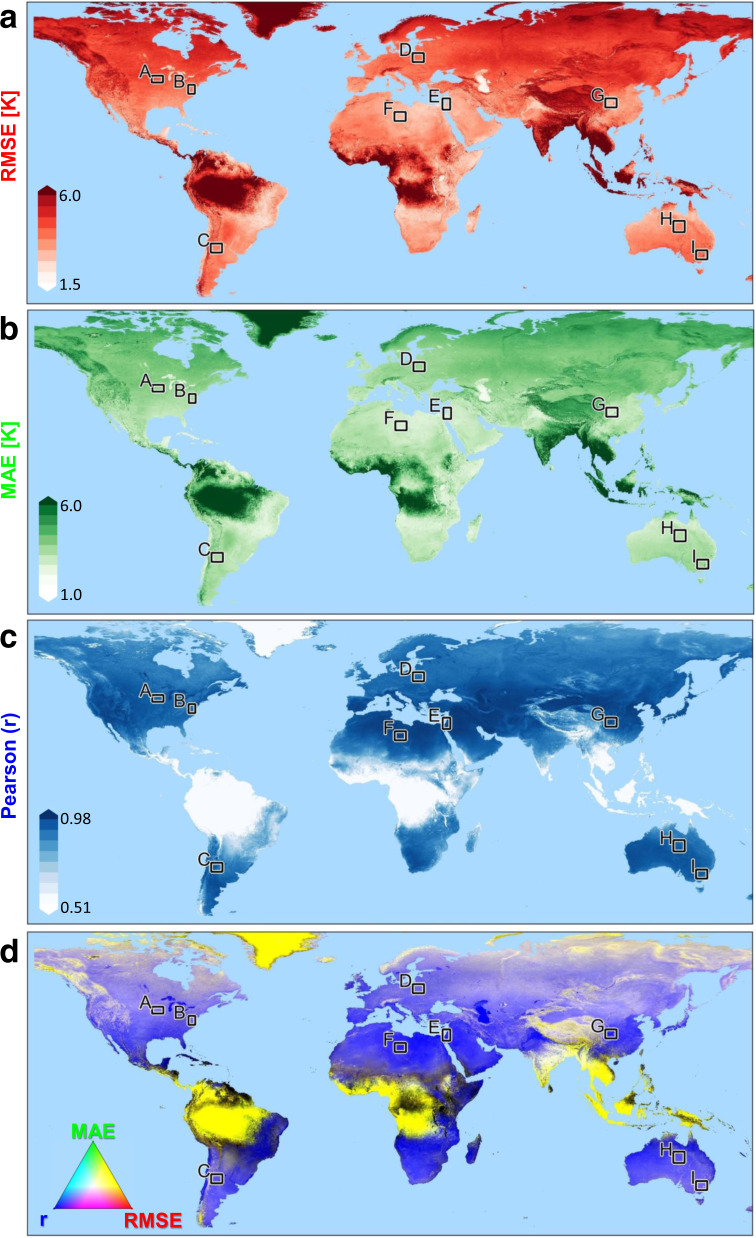
Same as Fig. 3, but for the entire world. The black rectangles represent the location of the nine global validation sites (Table 2), including the study area (E).

We provide the uncertainties (per pixel) by means of RMSE, MAE and Pearson correlation coefficient in our data set (as often done in such studies^41,42^). Figure 3 shows maps of RMSE (in red), MAE (in green) and Pearson r (in blue) in area E. RGB map of this three is also provided, showing areas with high RMSE and MAE and low Pearson r in yellow, areas with low RMSE and MAE and high Pearson r in blue. Generaly, the uncertainties are higher at higher altitudes which could be related to the effect of orographic clouds. Figure 4 shows maps of RMSE, MAE and Pearson r as in Fig. 3, but for the whole world. Areas with high cloud or snow frequencies are colored in yellow in Fig. 4d. We also provide a GEE code to calculate LST and the uncertainty metrics for areas of interest defined by the user. In addition, we provide a flagged dataset indicating whether the pixel’s data is original (observation) or gap filled. For the original data there are flags (provided by NASA/USGS) indicating 4 levels of uncertainty (i.e. less than: 1, 2, 3, and larger than 3 K)^28^.

## Usage Notes

The *LST*_*cont*_ dataset can be used for various applications and studies. For example this dataset is very usefull for different agricultural applications, such as optimization of decision making regarding crop location, timing, cultivar, and sowing. Furthermore, datasets for other regions can easily be produced by the GEE platform with the provided code or with the provided web application. Caution should be taken when running the code on regions with persistent cloudiness such as the equatorial regions. The variation in available data across the globe can be seen in Fig. 1c, and its effects on the uncertainties can be seen in Fig. 4.

**Fig. 5:**
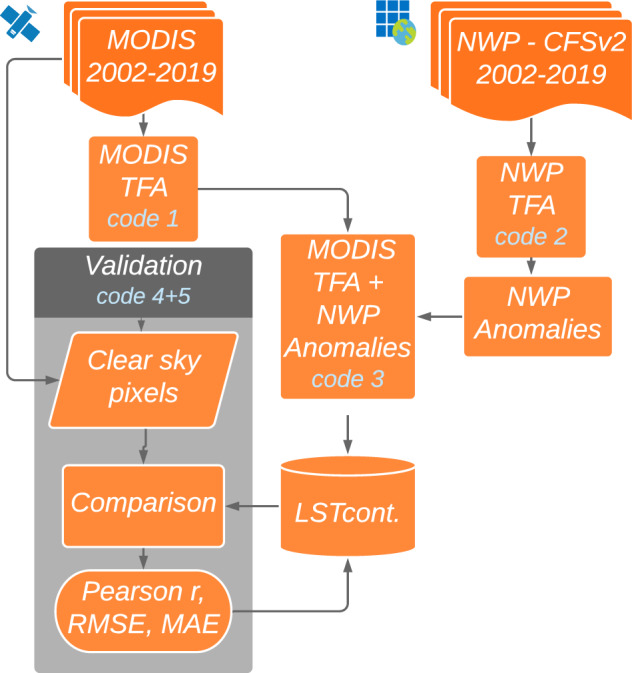
Flowchart of all the codes computing *LST*_*cont*_.

To produce *LST*_*cont*_ elsewhere, one can either use the *LST*_*cont*_ web application or reproduce the TFA (climatology) by using MODIS_TFA and CFSv2_TFA code files (codes 1 and 2 in Table 3) in a new area of interest. As described in Fig. 5, MODIS TFA and CFSv2 TFA should be available (by running the provided GEE code) before running the Continuous LST Export code file (code 3 in Table 3) to produce the final product – *LST*_*cont*_. Different code files have been prepared for day, night and daily mean LST datasets. All code files, including code files for validations (codes 4 and 5 in Table 3), are documented and available at GitHub (https://github.com/shilosh/ContinuousLST.git). A short movie on “How to visualize data using Qgis open source program” can also be found in the Github code repository.

**Table 3:** Inputs and outputs of the codes described in the usage notes and in Fig. 5.

Code	Input	output
**Code 1**: Prepare Satellite LST climatology	MYD11A1 (day, night)	LST TFA (day, night, daily)
**Code 2**: Prepare Model 2 m air temperature climatology	CSFv2 (00, 06, 12, 18 UTC)	CSFv2 TFA (12 UTC, daily)
**Code 3**: Prepare contLST	1. MYD11A1 (day, night) 2. LST TFA (day, night, daily) 3. CSFv2 (00, 06, 12, 18 UTC) 4. CSFv2 TFA (12 UTC, daily)	1. Day contLST 2. Night contLST 3. Daily contLST
**Code 4**: Create Performance Metrics for a selected area - (Yearly average)	1. MYD11A1 (day, night) 2. LST TFA (day, night, daily) 3. CSFv2 (00, 06, 12, 18 UTC) 4. CSFv2 TFA (12 UTC, daily)	1. RMSE 2. MAE 3. Pearson r
**Code 5**: Create (raster) Performance Metrics (per pixel for the selected area)	1. MYD11A1 (day, night) 2. LST TFA (daily) 3. CSFv2 (00, 06, 12, 18 UTC) 4. CSFv2 TFA (daily)	1. RMSE 2. MAE 3. Pearson r

The *LST*_*cont*_ web application (https://shilosh.users.earthengine.app/view/continuous-lst) is a Google Earth Engine app. The interface includes a map and a date picker. The user can select a date (July 2002 – present) and visualize *LST*_*cont*_ for that day anywhere on the globe. The web app calculate *LST*_*cont*_ on the fly based on ready-made global climatological files. The *LST*_*cont*_ can be downloaded as a GeoTiff with 5 bands in that order: Mean daily *LST*_*cont*_, Night original LST, Night *LST*_*cont*_, Day original LST, Day *LST*_*cont*_. In the dataset of the Eastern Mediterranean presented here the Day *LST*_*cont*_ is calculated based on both climatology and model anomalies as both products are almost synchronized in time, whereas the web app’s Day *LST*_*cont*_ is based solely on the climatology. The daily *LST*_*cont*_ is based on both climatology and model anomalies in the dataset as well as in the web application. Downloads via the web app interface are limited to areas smaller than 500,000 Km^2^ due to GEE limitations, nevertheless, GEE registered users can log in and download larger areas.

## Data Availability

GEE codes that calculates global *LST*_*cont*_ (*t*) and the validation of the dataset along with explanations on the usage of the code are publicly available through Github (https://github.com/shilosh/ContinuousLST.git) and Zenodo^43^ (10.5281/zenodo.3952603).
